# The PTSD help app in a Danish PTSD population: research protocol of a randomized controlled feasibility trial

**DOI:** 10.1186/s40814-020-00633-x

**Published:** 2020-06-30

**Authors:** Frederik Bernt Scharff, Marianne Engelbrecht Lau, Lisa Helena Grønberg Riisager, Stine Bjerrum Møller, Mehrak Lykkeberg Salimi, Matthias Gondan, Sofie Folke

**Affiliations:** 1 Unit for Psychotherapy Research, Psychotherapeutic Center Stolpegaard, Mental Health Services, Stolpegaardsvej 20, 2820 Gentofte, Capital Region of Denmark Denmark; 2 Hejmdal Private Psychiatric Hospital, Martinsvej 7-9, 1926 Frederiksberg C, Denmark; 3 Department for Military Psychology, Danish Veteran Center, Danish Defence, Svanemøllens Kaserne, Ryvangs Allé 1, 2100 Copenhagen, Denmark

**Keywords:** PTSD, mHealth, Feasibility

## Abstract

**Background:**

Due to an increase in PTSD patients seeking help in the Danish mental health sector and the addition of Complex PTSD to the ICD-11, there is a need to increase efficiency of existing treatments for PTSD. mHealth interventions have been shown to reduce PTSD symptoms. Therefore, the implementation of a mHealth intervention designed for psychiatric PTSD patients as a therapy add-on may improve treatment outcome. No study to date has explored the effects of mHealth interventions for PTSD in the Danish mental health sector, the feasibility and effect of this type of intervention needs testing.

**Methods:**

The study is an investigator-initiated randomized controlled feasibility trial investigating the clinical mHealth tool *PTSD help* combined with care as usual (CAU) compared to CAU for adults with PTSD. Seventy patients will be recruited and receive either the mHealth intervention combined with CAU or CAU alone. The primary feasibility outcome is the proportion of eligible patients that participate in the study until the end assessment. Secondary outcome data consists of the fraction of compliant patients in the experimental group and exploratory data on *PTSD help* on PTSD symptom severity, level of psychological distress, sleep quality, dissociation symptoms, therapy readiness, quality of life, disability levels, and recovery.

**Discussion:**

This study may help increase our knowledge of possible benefits of, as well as potential barriers to, the implementation of mHealth tools in the psychiatric sector. It may also provide a cost-efficient means to increase therapy outcomes and decrease the duration of suffering for PTSD patients in the psychiatric sector.

**Trial registration:**

The trial is registered at ClinicalTrials.gov (ID: NCT03862703) https://clinicaltrials.gov/ct2/show/NCT03862703 on the 27 of February 2019 and has been approved by the Danish Data Protection Agency (journal number: VD-2018-200 ISuite number 6443). Referring to the committee law §2, the National Committee on Health Research Ethics (DNVK) [H-18024180] decided that the study could proceed without approval as the use of *PTSD help* did not constitute a health science intervention according to Danish health science legislation.

## Background

Post-traumatic stress disorder (PTSD) is a potentially disabling and often protracted psychiatric disorder, which may develop as a response to one or several severely traumatic events. The prevalence of PTSD depends on several factors (e.g., the diagnostic system and assessment procedures), but across a range of European countries, the lifetime prevalence of PTSD is estimated to be approximately 2% [1]. The past years have seen a marked increase in the number of patients referred for PTSD treatment in Mental Health Services in the Capital Region of Denmark (MHS-CRD) from 453 in 2015 to 746 in 2017.

ICD-11 PTSD is comprised of three core symptoms: re-experiencing, avoidance, and hyperarousal (World Health Organization, 2018). In 2018, Complex PTSD (C-PTSD) was included in ICD-11 (World Health Organization, 2018). It contains the core PTSD components and an additional component reflecting “disturbances in self-organization” (DSO) comprised of affective dysregulation, negative self-concept, and disturbances in relationships [7, 18]. Furthermore, patients with C-PTSD are characterized by a higher frequency of childhood traumatic experiences and greater functional impairment [32] in comparison to PTSD patients. Cloitre et al. [20] report that C-PTSD is a predictor of drop-out and poor treatment response in some of the current first-line treatments for PTSD, and it has been suggested that this patient group may benefit from stabilizing interventions prior to trauma-focused psychotherapy [8, 17, 20]. Thus, there is a need to develop new treatment approaches that include stabilizing interventions for patients with C-PTSD.

Over the last decade, psychiatric treatment providers across the world have developed mobile technology both as standalone treatments and as supplements to existing treatments for mental health problems [5, 24]. The use of mobile health tools (mHealth) in mental health care is argued to have several potential advantages: mHealth tools can be easily integrated in daily life [4], they can reach people that do not normally seek mental health treatment [46], they can assist patients in getting psychoeducation [35, 40], and they can be used to monitor symptoms [14, 15, 35]. In addition, mHealth tools have the potential to improve the cost-effectiveness of interventions through the optimization of the clinicians’ time and resources and improve treatment effect by improving therapy engagement and adherence [14, 16, 21, 23, 48].

Existing clinical evaluations of mHealth tools have found promising indications of their efficacy in reducing patients’ symptoms and general level of psychological distress [27, 38]. Clinical studies investigating mHealth tools used as a psychotherapy add-on have also found enhanced compliance with treatment [45, 47], and increased treatment outcome, both immediately after end of treatment, and at 6-month follow-up, which may be partially due to an increase in patient adherence to homework [10, 39]. However, more research is needed to substantiate these results as most studies to date have not been replicated or have had methodological limitations such as a lack of control groups or small sample sizes [23, 38, 39, 43].

So far, mHealth apps for PTSD patients have primarily been designed for use by American veterans with PTSD [34, 44]. To address the needs of non-veteran PTSD patients, we developed an mHealth app, *PTSD help* (Danish: *PTSD hjaelp*), designed to supplement psychotherapeutic treatment for PTSD across psychotherapeutic modalities and PTSD subgroups. *PTSD help* focuses on psychoeducation (about PTSD symptoms, diagnosis, triggers, epidemiology, treatment, prognosis, aetiology, and advice for next of kin), emotion-regulating tools (e.g., breathing exercises, grounding exercises, calming images, body exercises, sleep advice, and guided meditations), crisis management (e.g., crisis plan), and finally, self-monitoring of PTSD symptoms and sleep quality. To ensure the usability of *PTSD help* across treatment modalities, the app does not include specific treatment elements as these are delivered in face-to-face psychotherapeutic treatment.

We have found no studies of mHealth tools for PTSD in a Danish treatment context, which makes it difficult to ascertain possible roadblocks for the implementation of mHealth tools in a Danish psychiatric context. However, a relatively broad implementation seems to be practically possible, as more than 85% of all Danes between 15 and 75 years own a smartphone. Because *PTSD help* is an untested clinical mHealth tool, it is necessary to test if it is possible to successfully recruit patient for an RCT testing efficacy of *PTSD help*, a Danish psychiatric context.

We therefore plan to conduct a feasibility study with the primary aim of ascertaining feasibility of randomization and investigate levels of patient compliance before conducting a larger randomized controlled trial (RCT) on treatment efficacy. The primary objective for the present study is to assess the feasibility of using *PTSD help* in the MHS-CRD to inform a larger future RCT study. The secondary objective is to gather preliminary exploratory data on the effectiveness of *PTSD help* versus treatment as usual on a range of clinically relevant outcome variables, including exploring C-PTSD as a potential moderator of subgroup-specific effects.

## Methods

The study is an investigator-initiated randomized controlled feasibility trial investigating *PTSD help* combined with care as usual (CAU) compared to CAU for adults with PTSD. The primary feasibility outcome is the proportion of eligible patients that participates in the primary app intervention before entering psychotherapy and provide data at the T1 (baseline) and T2 (post-app intervention) assessment. The secondary feasibility outcome is the fraction of compliant patients in the experimental group, and additional explorative outcome data consists of PTSD symptom severity, level of psychological distress, sleep quality, dissociation symptoms, therapy readiness, quality of life, negative events, and user satisfaction. This data will be collected using electronic questionnaires administered via participants email.

**Fig. 1:**
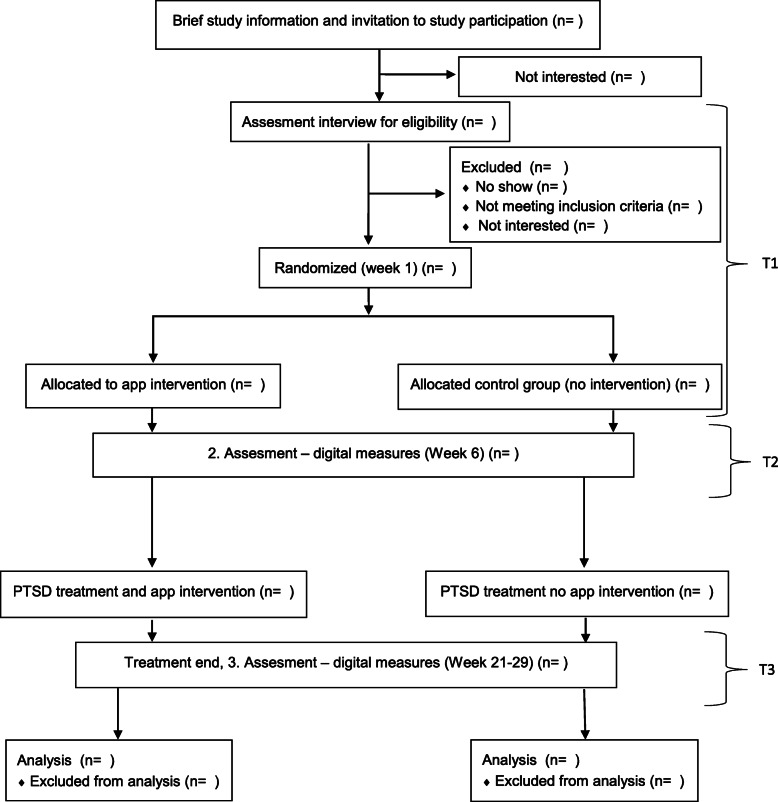
Consort diagram

The primary outcome will be assessed at T2 6 weeks after randomization. The secondary feasibility outcome will be assessed continuously by logging the patient’s interactions with the application. Secondary outcomes will be assessed prior to randomization and start of *PTSD help* intervention (T1), at post-primary *PTSD help* intervention (T2) and at the end of psychotherapeutic treatment (T3). The design of this trial has been developed with attention to the criteria outlined in the SPIRIT 2013 guidelines [13] (see Fig. 1 for a consort diagram).

The trial is registered at ClinicalTrials.gov (ID: NCT03862703) and has been reviewed by the Danish Data Protection Agency (journal number: VD-2018-200 ISuite number 6443). Referring to the committee law §2, the National Committee on Health Research Ethics (DNVK) [H-18024180] decided that the study could proceed without approval as the use of *PTSD help* did not constitute a health science intervention according to Danish health science legislation.

### Participants

The study will recruit patients referred with PTSD symptoms to the Centre for Visitation and Diagnostics (CVD) under the MHS-CRD, with the following inclusion criteria: the patient (1) must be at least 18 years old, (2) fulfil the DSM-5 PTSD diagnosis criteria, (3) be referred to PTSD care package treatment [22], (4) have access to a smartphone with iOS (Version 10 or higher) or Android (Version 5.0.1 or higher), and (5) provide informed consent. Exclusion criteria are patients with (1) suicidal risk, (2) ongoing episode of bipolar disease or psychotic disorder, (3) current abuse of alcohol or drugs, (4) inability to understand and/or read Danish, and (5) concurrent psychiatric or psychological treatment of PTSD outside of MHS-CRD.

### Randomization

Randomization is conducted through the data management software REDCap, which allows a setup where no one from the research team has knowledge of or access to the underlying computer-generated allocation sequence. The randomization uses permuted blocks with varying block sizes based on a computer-generated allocation sequence created by a researcher with no affiliation to the project. The randomization allocation ratio is 1:1. This procedure ensures that researchers are blinded to the allocation process as the randomization allocation list, and block size is unknown to the researchers. The randomization procedure will be stratified by presence of Complex PTSD as measured with the International Trauma Questionnaire [18].

### Blinding

Patients will be randomized after baseline assessment to ensure assessors and patients are blinded during the assessment. All assessments post-randomization will consist solely of self-report measures administered via email through REDCap and logging of user activity in the app.

### Procedure

Patients referred to CVD for diagnostic screening that are diagnosed with PTSD and accepted for PTSD outpatient treatment in MHS-CRD will be invited to participate in the study by the clinician conducting the screening. The invitation will include a leaflet with information about the study and contact information for the project. If the patient gives their consent for participation, he or she will be booked for an assessment interview. Patients who provide written consent and complete the assessment interview will be randomized if they fulfill the inclusion/exclusion criteria. After randomization, patients in the experimental group will be given immediate access to *PTSD help.* After 3 days, they will receive a phone call to ascertain whether they are experiencing any technical difficulties using the app. The second and third assessments will be administered electronically via a link to the questionnaires sent in an email through REDCap (see Fig. 1 for a consort diagram).

### Interventions

#### PTSD treatment

All patients in the project will receive PTSD treatment at one of six outpatient clinics in the MHS-CRD that provides PTSD treatment. All clinics comply with official Danish recommendations for PTSD Care Packet-treatment (CP-treatment) [22]. PTSD CP-treatment consists of 60 h trauma-focused group psychotherapy or 15 h trauma-focused individual therapy combined with other treatment elements such as assessment, psychoeducation, pharmacological counselling/treatment, social skill training, meeting with social network, and monitoring. The CP includes 75 h of treatment in total (Table 1).

**Table 1:** Care package treatment for post-traumatic stress disorders

CP content	hours
Psychiatric and somatic assessment	3 h
Psychometry (monitoring) and psychoeducation	2 h
Group psychotherapy	60 h
Psychopharmacology	2 h
Social skills training and couple counselling	4 h
Continuity and coherence in ongoing treatment	4 h
Total	75 h

#### PTSD help

The *PTSD help* app is designed for use in preparation for and as a supplement to psychotherapy. The app includes a range of different functions. It contains psychoeducation about PTSD on a range of different topics including PTSD symptoms, etiology, prognosis, treatment, trauma in general, and information for next of kin on how to offer support to individuals suffering from PTSD. The app contains tools for self-assessment and monitoring of PTSD symptoms (*PTSD checklist for DSM-5,* [53]) and sleep quality (*Sleep Condition Indicator*, [25]). For alleviating sleep problems, the app has two different audio-recorded guided meditations and advice for improving sleep habits and sleep environment. It also contains different tools and techniques for relieving anxiety such as distraction exercises, a breathing exercise with an animated prompt to help the user regulate his or her breath, physical relaxation and simple yoga exercises, calming images accompanied with music, and an option for taking personal notes on the experience of specific symptoms and helpful strategies to reduce distress associated with these symptoms. And finally, it has a crisis plan with personal contacts and contact information for psychiatric emergency services. The features selected for the app were chosen based on advice from resident clinical experts and empirical evidence drawn primarily from the cognitive behavioral tradition of psychoeducation and anxiety management training [28, 29] and from cognitive behavioral treatment for insomnia [11, 51]. In addition, some features based on yoga and meditation practices were included, based on emerging evidence for their potential effectiveness [30, 36]. *PTSD help* was freely available to participants in the experimental group, but is currently not broadly available for download. The current build of the app will be kept stable throughout the intervention.

### Assessment interview

The patient’s trauma history is assessed with *The Trauma History Questionaire* [31], a 24-item self-report measure of the patient’s lifetime trauma history. PTSD diagnosis and comorbid disorders are assessed through the *Mini International Neuropsychiatric Interview* (MINI v. 7.02) [50], a structured diagnostic interview that assesses the presence of psychiatric disorders according to DSM-5. To ascertain whether the patient also fulfills the criteria for *International Classification of diseases 11*^*th*^*revision pending* (ICD-11) PTSD or C-PTSD, the *International Trauma Questionaire* (ITQ) [19] is administered. ITQ is a 23-item self-report questionnaire used to distinguish between PTSD and C-PTSD as defined in ICD-11.

### Feasibility outcome

The primary feasibility outcome is the proportion of eligible patients that agree to be randomized and participate in assessment 1 (T1, week 1) and 2 (T2, week 7). The secondary feasibility outcome is the fraction of compliant patients in the experimental group that is patients that actively use the system after. Compliance is defined as the use of *PTSD help* functions (excluding self-monitoring tools) corresponding to use twice a week (mean), assessed over 6 weeks pre-treatment, and during the psychotherapy treatment period. Compliance data is collected from the tracking log of the patient’s app activity through a secured webpage. In addition, compliance will be assessed through a *user behaviour questionnaire,* an 11-item questionnaire developed by the project group for the current study by selecting and modifying relevant items from questionnaires used in earlier studies evaluating the *PTSD Coach* app [34, 44] (see Additional file 1).

### Exploratory outcomes

Exploratory outcomes in the study are app intervention dropout rate, psychotherapy dropout rate, and a range of patient outcome variables measured with the following questionnaires (Table 2). PTSD symptoms are measured using the *PTSD Checklist for DSM-5* (PCL-5) [53], a 20-item self-report questionnaire that assesses the presence of the four core DSM-5 PTSD symptom clusters during the past month. The *Dissociative Symptoms Scale* (DSS) [12] is used to measure the presence of dissociative symptoms. DSS is a 20-item self-report questionnaire measuring four domains of dissociation: depersonalization, derealization, gaps in awareness or memory, and dissociative re-experiencing. The patient’s general level of psychological distress is measured with *the Symptom Checklist-10* (SCL-10) [3], a 10-item measure of general psychological distress, derived from the *Symptom Checklist-90* (SCL-90). SCL-10 is composed of questions that measure the three factors of the SCL-90 that accounts for the largest amount of variance: depression, somatization, and phobic anxiety. *WHO-5* [2] is used to measure the patients’ quality of life. WHO-5 is a 5-item self-report questionnaire that measures well-being during the last 2 weeks. Patient sleep quality is measured with the *Sleep Condition Indicator* (SCI) [25, 37], an 8-item questionnaire. The patient’s readiness to change is measured with *The University of Rhode Island Change Assessment Short version* (URICA-S) [41], a short version of the URICA self-report questionnaire [42]. URICA-S is a 16-item measure. Possible negative effects of the *PTSD help* intervention are measured with the *Negative Events and Results of Psychological Treatment–Revised* (NEQ-rev) [31, 49], a 13-item self-report questionnaire that aims to assess negative effects associated with psychotherapeutic interventions, e.g., suicidal thoughts, lower self-esteem, increased stress level, and worsening of symptoms. NEQ-rev is a revised version of NEQ. All items in NEQ that directly concern treatment-related conditions or outcomes of psychotherapy are excluded (see Additional file 2 for a list of excluded items), as the patients do not undergo psychotherapeutic treatment at the time NEQ-R is administered. To measure user experiences, *the user satisfaction questionnaire* was developed by the project group. This is a 17-item self-report questionnaire that assesses the patient’s perceived benefit from using the app *PTSD help*. The project group developed the questionnaire selecting, translating, and modifying relevant items from two questionnaires used for evaluation of the *PTSD Coach* app by Kuhn et al. [33] and Miner et al. [44] (see Additional file 1).

**Table 2:**
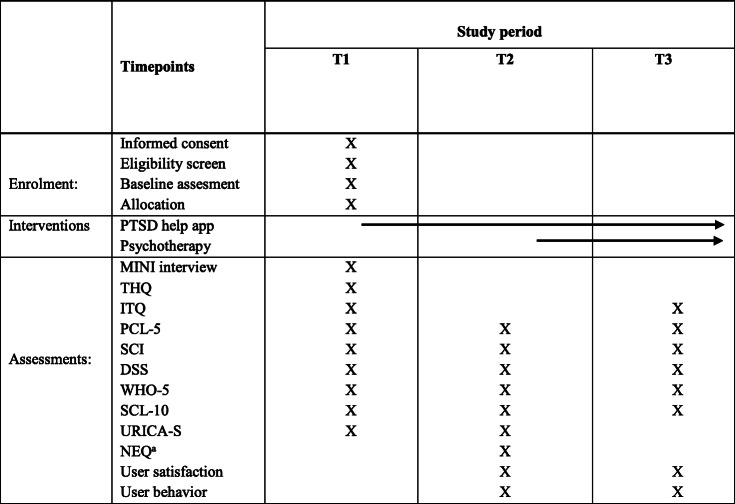
SPIRIT figure

*T1* enrollment, baseline assesment, and randomization, *T2* post-primary app intervention 6 weeks after baseline, *T3* end of psychotherapy treatment, *MINI* Mini**-**International Neuropsychiatric Interview**,***THQ* Trauma History Questionnaire, *I* International Trauma Questionnaire, *PCL-5* PTSD Checklist for DSM-5, *SCI* Sleep Condition Indicator, *DSS* Dissociative Symptoms Scale, *WHO-5* WHO-Five Well-being Index, *SCL-10* Symptom Checklist-10, *URICA-S* University of Rhode Island Change Assessment Scale-Short, *NEQ* Negative Effects Questionnaire, *Feasibility and Acceptability Questionnaires* developed specifically for this project by the research team

^a^Revised version of NEQ, 13 questions selected

### Adverse events

As the interventions included in the app does not include any trauma-focused interventions or other symptom provoking interventions, we do not expect any adverse effect from use of the app. However, to ascertain whether this assumption is correct, we include the *Negative Events and Results of Psychological Treatment–Revised* to measure possible adverse effects.

### Statistical analysis

The primary and secondary outcomes are analyzed through descriptive statistics, e.g., percentage of patients that show up at T2, the average usage of the system over the intervention period, and the proportion of patients that stay in study until the end.

Analysis of between-group effects on explorative outcome measures will be analyzed with analysis of covariance, using the intervention arm as the main predictor and baseline severity and the stratification variable from randomization as covariates. For binary outcomes (e.g., compliance), logistic regression will be used. Due to the exploratory nature of this feasibility study, missing data will not be imputed but the amount of missingness will be reported. Results will be presented as covariate adjusted group differences or odds ratios, along with the 95% confidence interval. If the results indicate feasibility, we will proceed to a randomized controlled efficacy study.

### Sample size

Due to a shortage of similar studies, it is not currently possible to conduct a calculation of optimal sample size for this study. Based on existing knowledge about feasibility study methodology [6, 52, 54] and two feasibility studies of mHealth tool in public health settings [9, 26], we plan to recruit 70 patients. With 70 patients, the 95% confidence interval for a proportion estimate such as compliance would range between 0.38 and 0.62 (if the observed proportion is 0.50), which we consider acceptable for planning further studies.

## Discussion

The aim of this trial is to investigate the feasibility of implementing a large scale RCT of the *PTSD help* app and to generate hypotheses about the effectiveness of *PTSD help* for psychiatric patients suffering from PTSD in a Danish psychiatric setting. The results of this trial will be used to inform a potential RCT by providing data for sample size estimation, optimizing the recruitment procedure with regards to the exclusion and inclusion criteria, identifying roadblocks for patients use of the app and supplying information about potential modification to the content of the app, both through analysis of the patients app use patterns, and responses on the user behavior questionnaires. In addition, as this is, to our knowledge, the first trial of a mHealth supplement for PTSD treatment is in a Danish population. This study may help increase our knowledge of possible benefits of, as well as potential barriers to, the implementation of mHealth tools. In the future, this may assist the development and implementation of mHealth tools for a range of psychiatric disorders. This may have the potential to increase therapy outcomes and decrease duration of suffering and the costs of treatment in the Danish psychiatric sector*.* In addition, this study can provide information on the effect of providing interventions in the pre-treatment period, as opposed to the waiting list condition. The measurements of clinical variables in this time period will also provide information on the effect of the pretreatment period.

As the study is a feasibility study, it has several limitations. First and foremost, due to the small sample size, it will be impossible to test the clinical effect on the various outcome measures. However, the relatively small sample size is deemed adequate to determine feasibility, which is the primary goal of the study. An additional limitation is that the study setting has made it impossible to create homogenous experimental and control therapy groups in the group setting, in that there will be a mix of patients from the two conditions in the therapy groups. Another limitation due to the study setting is that patient may receive a relatively wide variety of pharmacological treatments at different time points during the trial, information about which is not available to the research group due to GDPR rules limiting access to patient medical information. This lack of consistency in pharmacological treatment across patients limits the possibility to assess the direct impact of the app intervention, though this is partially mitigated by randomization. A general limitation in studies including mHealth tools in a psychiatric setting is that even though a large majority of adults have access to a smartphone, some do not, which will limit possible intake applicability of mHealth tools.

## Supplementary information

**Additional file 1.** PTSD help user behaviour.

**Additional file 2.** Excluded NEQ items.

## Data Availability

The data of this study are available on request from the corresponding author FBS.

## Abbreviations

C-PTSD: Complex PTSD
DSM-5: Diagnostic and Statistical Manual of Mental Disorders, Fifth Edition
DSS: *Dissociative Symptoms Scale*
ICD-11: International Classification of Diseases 11th Revision
IPT: International Trauma Questionnaire
MHS-CRD: Mental Health Services, Capital Region Denmark
MINI: *Mini International Neuropsychiatric Interview*
NEQ-rev: *Negative events and results of psychological treatment–revised*
PCL-5: *PTSD checklist for DSM-V*
PTSD: Post-traumatic stress disorder
RCT: Randomized controlled trial
SCI: *Sleep Condition Indicator*
SCL-10: *The Symptom Checklist-10*
*SCL-90*: The Symptom Checklist-90
SDS: Sheehan Disability Scale
SPIRIT: Standard Protocol Items: Recommendations for Interventional Trials
URICA-S: *The University of Rhode Island Change Assessment Short version*
WHO-5: The WHO-Five Well-being Index
