# Supplementary material for: The PTSD help app in a Danish PTSD population: research protocol of a randomized controlled feasibility trial
Source: Pilot Feasibility Stud. 2020 Jun 30;6:92. doi: 10.1186/s40814-020-00633-x (PMC7325563; doi:10.1186/s40814-020-00633-x)
Supplement: Supplementary file 1 — Additional file 1. PTSD help user behaviour. [file 40814_2020_633_MOESM1_ESM.docx]

# Appendix 1

# PTSD help user behaviour

In this questionnaire we would like to ask about your use of the PTSD help app.

1. On average, how many times a week have you used the app?

0-1minutes

1-3 minutes

3-5 minutes

5-10 minutes

10-20 minutes

More than 20 minutes

1. How many minutes have you typically spent on the app

each time?

Learn about PTSD

Personal notes and strategies

Crisis plan

Favorites

PTSD test

Sleep quality test

10 pieces of advice for better sleep

Calming images

Distraction exercises

Help for falling asleep

Body exercises

Pleasurable activities

Take a deep breath

1. Which pages in the app have you used the most?

(you may mark more than one option)

At home

At work or in school

When I had to fall asleep

During the night

In stressful situations (e.g. on the bus, on the train, at social events)

Before treatment sessions

1. What places and in which situations have you

typically used the app? (you can check off more than

one option)

Before

1. Have there been other places or situations where you have typically used the app? If so, where?

## Have you experienced that the following factors have limited your use of the app?

1. Not enough time.
2.
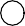

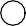

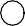

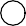
The app was hard to use.
3.
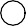

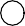

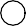

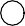
I didn't get much from using the app.
4.
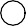

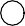

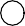

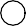
I couldn't find what I needed.

Wasn't a problem Was a small problem Was a big problem Was a very big

problem


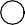

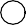

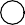

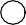


# PTSD help user satisfaction

Now we would like to ask you about your experience with using the PTSD help app.

## How useful were the following functions in the app?:

1. Learn about PTSD?
2. Personal notes on symptoms and strategies?
3. Crisis plan?
4. Favorites?
5. PTSD test?
6. Sleep quality test?
7. 10 pieces of advice for better sleep?
8. Calming images?
9. Distraction exercises?
10. Help for falling asleep?
11. Body exercises?
12. Pleasurable activities?
13. Take a deep breath?

Not at all A little Somewhat Very Extremely


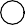

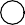

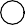

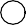

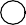


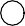

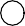

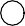

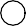

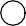


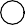

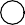

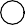

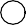

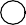

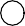

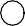

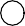

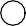

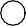

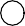

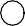

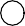

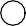

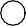

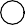

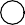

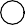

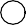

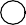


Not at all A little Somewhat Very Extremely


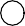

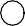

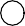

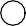

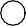


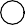

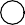

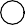

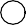

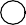

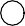

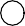

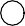

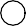

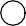

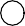

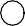

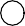

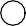

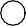

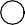

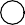

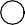

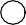

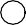

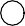

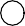

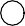

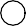

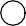

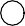

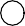

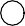

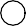

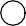


## To what extent do you agree with the following:

1. The app has increased my knowledge of PTSD.
2.
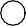

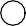

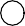

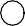

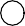
The app has helped me better understand PTSD symptoms.

Disagree Disagree somewhat


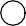


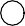

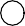


Either or Agree somewhat Agree


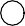

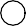


1. The app has helped me
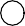

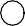

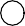

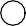

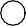
 follow changes in my symptoms.
2. I can use the tools in the
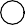

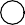

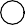

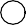
 app to regulate my emotions.
3. Through the app I have learned new ways to handle my

symptoms.

19) Using the app has made me feel that I can do something about my PTSD.

20) Using the app has helped

me talk about my experiences.

## How do you rate the app in general?

Not at all A little Somewhat Very Extremely

1. How easy was it to use the app compared to for example

obtaining information on paper?

1. In general, how satisfied were you

with the PTSD help app?

1. How was the app most useful to you? (describe below)

# PTSD hjælp brugeradfærd

I dette spørgeskema vil vi gerne spørge dig til din brug af PTSD hjælp appen.

1. Hvor mange gange om ugen har du i gennemsnit

brugt appen?

0-1 minut

1-3 minutter

3-5 minutter

5-10 minutter

10-20 minutter

Over 20 minutter

1. Hvor mange minutter har du typisk brugt appen pr.

gang?

Lær om PTSD

Personlige noter og strategier

Kriseplan

Favoritter

PTSD test

Søvnkvalitetstest

10 råd til bedre søvn

Beroligende billeder

Distraktionsøvelser

Hjælp til at falde i søvn

Kropsøvelser

Lysbetonede aktiviteter

Tag en dyb indånding

1. Hvilke sider i appen har du brugt mest?

(du må gerne markere mere end én mulighed)

Hjemme

På arbejde eller i skole

Når jeg skulle falde i søvn

Om natten

I stressende situationer (f.eks. i bussen, i toget, til social arrangementer)
Før behandlingssamtaler

1. Hvilke steder og i hvilke situationer har du

typisk brugt appen? (du må gerne markere mere end én

mulighed)

1. Har der været andre steder eller situationer hvor du typisk har brugt appen? Hvis ja, hvilke?

## Har du oplevet, at nogle af følgende ting har begrænset din brug af appen?

1. Ikke nok tid.
2. Appen var svær at bruge.
3. Jeg fik ikke ret meget ud af at bruge appen.
4. Jeg kunne ikke finde dét, jeg havde brug for.

Var ikke et problem Var et lille problem Var et stort problem Var et meget stort problem

# PTSD hjælp brugertilfredshed

Her til sidst vil vi gerne spørge til din oplevelse af at bruge PTSD-hjælp appen.

## Hvor brugbare var de følgende funktioner i appen?:

1. Lær om PTSD
2. Personlige noter om symptomer og strategier
3. Kriseplan
4. Favoritter
5. PTSD test
6. Søvn kvalitetstest
7. 10 råd til bedre søvn
8. Beroligende billeder
9. Distraktionsøvelser
10. Hjælp til at falde i søvn
11. Kropsøvelser
12. Lystbetonede aktiviteter
13. Tag en dyb indånding

Slet ikke Lidt Nogenlunde Meget Rigtigt

meget/ekstremt

Slet ikke Lidt Nogenlunde Meget Rigtigt

meget/ekstremt

## Hvor enig er du i følgende udsagn?

1. Appen har forbedret min viden om PTSD.
2. Appen har hjulpet mig til bedre at forstå PTSD symptomer.
3. Appen har hjulpet mig til at kunne følge med i ændringer af mine symptomer.Meget uenig Uenig Hverken enig

eller uenig

Enig Meget enig

1. Jeg kan bruge redskaberne fra

appen til at håndtere mine

følelser.

1. Jeg har via appen lært nye

måder, som jeg kan håndtere mine symptomer på.

1. Det at bruge appen, har fået

mig til at føle, at jeg selv

kan gøre noget ved min PTSD.

1. Det at bruge appen, har hjulpet

mig til at tale om det, jeg

har oplevet.

## Hvordan vurderer du generelt appen?

1. Hvor nemt var det at bruge appen, sammenlignet med eksempelvis at få information på papir?

Slet ikke Lidt Nogenlunde Meget Rigtigt

meget/ekstremt

1. Overordnet set, hvor tilfreds er

du med PTSD hjælp appen?

1. På hvilken måde var appen mest brugbar for dig? (beskriv her)
