# Supplementary material for: The PTSD help app in a Danish PTSD population: research protocol of a randomized controlled feasibility trial
Source: Pilot Feasibility Stud. 2020 Jun 30;6:92. doi: 10.1186/s40814-020-00633-x (PMC7325563; doi:10.1186/s40814-020-00633-x)
Supplement: Supplementary file 2 — Additional file 2. Excluded NEQ items. [file 40814_2020_633_MOESM2_ESM.docx]

Appendix 2 – Excluded NEQ items

1. Jeg føler, at jeg er blevet afhængig af min behandling
2. Jeg forstod ikke altid min behandling
3. Jeg forstod ikke altid min terapeut
4. Jeg havde ikke tiltro til min behandling
5. Jeg følte, at behandlingen ikke gav noget resultat
6. Jeg oplevede ikke, at mine forventninger til terapeuten blev indfriet
7. Jeg synes, at behandlingen var umotiverende
8. I feel that I have become dependent on my treatment
9. I didn’t always understand my treatment
10. I didn’t always understand my therapist
11. I had no confidence in my treatment
12. I felt that the treatment didn’t produce any results
13. I didn’t find that my expectations of the therapist were met
14. I feel that the treatment was demotivating
